# How has the presidential election affected young Americans?

**DOI:** 10.1186/s13034-018-0214-7

**Published:** 2018-02-13

**Authors:** Melissa DeJonckheere, Andre Fisher, Tammy Chang

**Affiliations:** 1 Department of Family Medicine, University of Michigan, 1018 Fuller St, Ann Arbor, MI 48104 USA; 2 University of Michigan, 500 S. State St, Ann Arbor, MI 48109 USA; 3 Institute for Healthcare Policy and Innovation, University of Michigan, 2800 Plymouth Rd, Ann Arbor, MI 48109 USA

**Keywords:** Adolescents, Mental health, Stress

## Abstract

The 2016 presidential election season and subsequent political events have had physical and emotional impacts on youth. We collected qualitative insights from 14 to 24 year olds across the US related to these events over time. Open-ended probes were sent via text message at three time points before and after the 2016 presidential election. The majority of youth reported emotional stress during all three time points, and female participants were significantly more likely to experience emotional responses. White participants were more likely to report negative symptoms than their peers both pre-election and at 4-months post-election. While preliminary, the results indicate that feelings of stress, anxiety, and fear have persisted in the months following the election, particularly for young women. Additional research is needed to examine the long-term effects of political events on the emotional and physical health of youth.

## How has the presidential election affected young Americans?

This past presidential election season and subsequent political events have affected many adults in the US both emotionally and physically [1], yet the impact on young people is not well understood. Research supports that current events, including election results, can impact health outcomes [2, 3]. For example, following the attacks on September 11th, health outcomes for Arab Americans worsened including lowered life expectancies and increased rates of premature births. Following the 2008 election, supporters of the losing candidate had higher cortisol responses than their peers. In both examples, political events worsened levels of stress and anxiety. In the months leading up to and following the 2016 presidential election, countless editorials and articles in popular media detailed the negative impact of the election season on US adults across political affiliations and states. To our knowledge, this phenomenon has not been adequately investigated in adolescents or young adults.

Stress in youth is not benign and is linked to poor emotional and physical health effects. Specifically, stress is linked to cardiovascular disease, anxiety, depression, aggression, substance abuse, and behavioral problems in adolescence, as well as poor outcomes in adulthood [4–6]. The outcomes associated with stress are worsened when events are uncontrollable, like politics may feel to youth, leaving them feeling hopeless and unable to cope with adversity. As youth increasingly rely on social media for news and communication, the constant presence of information related to the election and subsequent political events may further impact the influence on youth.

We gather data from youth throughout the US every week via text messaging on a wide variety of health-related topics (see research protocol for description of ongoing study) [7]. Weekly topics reflect timely youth-related policy issues or health concerns. Eligible participants (14–24 years of age, access to a phone with SMS capabilities) are recruited at community events and through social media, consented, and sent a link to an online demographic questionnaire. To understand youth experiences during and following the election, we used a longitudinal mixed methods design with three time points: 1-week pre-election, 2-weeks post-election, and 4-months post-election. Qualitative probes were developed to be open-ended and elicit narrative responses about participant beliefs, attitudes, and behaviors during the election season. Qualitative data were imported into MAXQDA software for analysis. Two researchers coded the data through a process of open coding and discrepancies were discussed until consensus was reached. Responses were then categorized as negative, neutral, or positive for quantitative descriptive analysis and compared using t-tests.

80 participants responded to at least one of the probes (pre-, post-election, or 4-months post-election) and had a mean age of 18.3 (SD = 2.53; Table 1). Of those who completed the optional demographic survey (n = 69), nearly half were women (49%). The majority identified as White (49%; Black 15%, Asian 14%, Latino 4%) and most had completed at least some high school (49%) or some college (25%). Evidenced by responses to open-ended probes, our sample included youth who supported candidates across the political spectrum.

**Table 1:** Participant demographics n = 80

Characteristic	
Age, M (SD)	18.3 (2.53)
Gender, n (%)	
Female	39 (49)
Male	28 (35)
Genderfluid or trans	2 (3)
Unspecified	11 (14)
Race and ethnicity, n (%)	
White or Caucasian	39 (49)
Black or African American	12 (15)
Asian or Pacific Islander	11 (14)
Latino or Hispanic	3 (4)
American Indian or Alaska Native	2 (3)
Multiple races	5 (6)
Unspecified	11 (14)
Education, highest level completed, n (%)	
Some high school	31 (39)
High school graduate	8 (10)
Some college	20 (25)
College graduate	4 (5)
Some graduate school	2 (3)
Graduate degree	2 (3)
Unspecified	13 (16)

Our results showed that a large proportion of youth were affected by the election, either emotionally (86% pre-election; 71% post-election; 63% 4-months post-election) and/or physically (20% pre-election; 19% post-election). Female participants were more likely to experience emotional responses at all three time points (82% pre-election; 80% post-election; 51% 4-months post-election; p < .05). Male participants reported emotional symptoms, but with less prevalence (61% pre-election; 50% post-election; 32% 4-months post-election). White participants were more likely to report negative symptoms than non-white participants both pre-election and 4-months post-election (p < .05). For those that reported stress pre-election, negative symptoms remained 4-months after the election (see Table 2 for representative qualitative data).

**Table 2:** Representative quotes from qualitative probes

Participant characteristics	Pre-election	2-weeks post-election	4-months post-election
White, female, 15, some high school	It makes me lose faith in Americans. It makes me afraid to be myself because it made me afraid of racist people. It makes me fear the future and makes me want to not live in the US anymore	It makes me scared for my safety. It makes me think that in the future Americans will think it’s ok to discriminate against minorities. I’m worried that when wearing a hijab, that me or my mom will be attacked verbally or physically	The travel ban affected me greatly. I’ve felt upset and frankly a little scared for me and my family’s safety. [The result] made me involved in the news and made me voice out my opinion as much as I can
White, male, 16, some high school	In many conversations this election sneaks it’s way in, everyone seems to have a strong opinion and seems to hate the other side. This can be very stressful	I definitely am not as personally targeted as other individuals, however I still feel quite a lot of fear. The cabinet is starting to materialize the worst scenarios	I’ve felt terrible for those who are more directly negatively affected by Trump’s policies, and rather guilty that they would benefit my family, which I don’t want
Black, male, 17, some high school	I don’t really care. I think my future will be a bit more cloudy	[The election result] hasn’t affected me at all	Don’t care, not one bit
White, genderfluid, 20, high school graduate	[The election has] made me really anxious… Anxiety leads to me not sleeping as well	I’m really scared and upset… harder to sleep… [I feel] more scared, feel like the future might look like a dystopian novel	I had already been doing activist work, but it got me involved in some new groups
Asian, female, 20, some college	It has made me shocked and frustrated and disappointed. It has made me more pessimistic about the future	I’ve felt shocked and frustrated. I’m tired. I’m worried about the availability of options for my future and being discriminated against because of my religion	I’ve felt upset, frustrated, sad. [Since the election] I’ve tried to be more compassionate toward others no matter what. You don’t know what people are going through
Asian, male, 20, college graduate	Hasn’t affected me too much emotionally or physically either. I can feel some increased stress buildup in my shoulders if I think about the future of America for too long	I am much more hesitant and worried about my future as well as America’s future as a whole. However, I am a hopeful person and strongly believe that everything will be ok	I have been saddened by our country’s choices. It seems like racism and violence are on the rise, and the rich and powerful have found their greatest ally in our government. I’ve given up and just hope people make it out alive
White, female, 21, some college	I feel stressed because of how intense and effectual [the election] could be. I feel depressed about the future	I feel as though my role as a woman in society was not validated and MOST people are not good people, valuing themselves more than others. I’m not sleeping some nights, adding stress, increasing cortisol levels. I feel as though the world is not improving and getting better, but instead moving backwards	I have been doing so poorly [emotionally and physically]. It has been so stressful, so I go on Facebook less
White, female, 23, some graduate school	It’s been extremely frustrating to constantly hear the normalized hateful rhetoric directed towards women, racial/ethnic minorities, and specific religious groups. It’s contributed to my stress levels, which has made me feel more overwhelmed with my responsibilities with school and work–my poor coping mechanisms involve stress eating and exercising less	I’m worried he will harm refugees and immigrants already in the country and those who will try to enter. I’m worried that he will repeal the health insurance laws that provided insurance to many people. I’m worried that he will limit access to women’s health care. I’m worried that he will make poor foreign affairs decisions that will compromise the security of people in the US and around the world	I feel upset and overwhelmed. I’ve disconnected from the news a lot recently which has helped

The most commonly reported emotional responses throughout this time period were stress and anxiety (“It’s contributed to my stress levels, which has made me feel more overwhelmed with my responsibilities”), worry (“I’m worried that [xenophobia] will continue to escalate after the election”), fear (“I am scared honestly, I don’t know what is going to happen”), and disappointment (“It’s upsetting that [the election] is such a mess”). Physical symptoms included feeling “tired,” “drained,” and “nauseous.” One individual wrote that she was “not sleeping some nights” because of the election, while another described how hard it was to concentrate and do well in school after the election. Stress affected vocal supporters of both candidates and vocal supporters of neither candidate. The small proportion who did not experience any emotional or physical symptoms across time points wrote that the election “hasn’t affected” their daily lives or that they “don’t worry about politics.”

The election season and subsequent political events have already had emotional and physical repercussions on youth, making it an ongoing major public health concern that must be addressed. While preliminary and exploratory, the results of this longitudinal mixed methods study indicate that feelings of stress, anxiety, and fear have persisted in the months following the election, particularly for young women. We will continue to explore the impact of political events on youth in the US through ongoing quantitative and qualitative data collection. This research adds to the limited evidence that election results can impact emotional and physical health [1–3], though more research is needed to investigate this phenomenon in a representative sample of youth and to examine the long-term effects.

## References

[1] American Psychological Association. Stress in America: US Presidential election. http://www.apa.org/news/press/releases/2016/10/presidential-election-stress.aspx. Accessed 12 Sept 2017.

[2] Lauderdale DS (2006). Birth outcomes for Arabic-named women in California before and after September 11. Demography.

[3] Stanton SJ, Labar KS, Saini EK, Kuhn CM, Beehner JC (2010). Stressful politics: voters’ cortisol responses to the outcome of the 2008 United States presidential election. Psychoneuroendocrinology..

[4] Grant KE, Compas BE, Thurm AE, McMahon SD, Gipson PY (2004). Stressors and child and adolescent psychopathology: measurement issues and prospective effects. J Clin Child Adolesc Psychol..

[5] Low CA, Salomon K, Matthews KA (2009). Chronic life stress, cardiovascular reactivity, and subclinical cardiovascular disease in adolescents. Psychosom Med.

[6] Romeo RD (2013). The teenage brain: the stress response and the adolescent brain. Cur Dir Psychol Sci..

[7] DeJonckheere M, Nichols LP, Moniz M, Sonneville KR, Zhao X, Vydiswaran VG, Guetterman TC, Chang T (2017). MyVoice national text message survey of youth aged 14–24 years. JMIR Res Protoc..

